# A Curious Case of Hemolytic Anemia with Pseudoreticulopenia

**DOI:** 10.1155/2022/6423143

**Published:** 2022-04-21

**Authors:** Scott Mayer, Nikhilesh Srinivasan, Jenny Nguyen, Ryan Spilman, Dmitriy Scherbak

**Affiliations:** ^1^HCA HealthONE, Lone Tree, CO, USA; ^2^Rocky Vista University School of Osteopathic Medicine, Lone Tree, CO, USA

## Abstract

Herein, we present a unique case of a Coombs-negative, steroid-refractory autoimmune hemolytic anemia (AIHA) complicated by pseudoreticulopenia, describe its clinical presentation, histopathologic findings, and management, and review the salient literature. Coombs-negative, steroid-refractory AIHAs represent fewer than 1% of all AIHAs. Diagnosis of the disease is difficult and often delayed due to the pursuit of alternate diagnoses following a negative Coombs test. However, when suspicion remains high for an autoimmune process, the super-Coombs test may be utilized for the diagnosis of AIHA that the traditional Coombs test fails to detect. A majority of cases respond to rituximab as the indicated second-line therapy, but delays in diagnosis and subsequent treatment may increase morbidity. Reticulopenia may be associated with AIHAs secondary to bone marrow dysfunction, but this patient had a normal function marrow confirmed on biopsy. Indeed, reticulopenia in this case was a diagnostic conundrum that further obscured the diagnosis and delayed treatment. Ultimately, reticulopenia was determined to be pseudoreticulopenia secondary to an alteration in the maturation of the erythroid lineage due to an independent, newly diagnosed pernicious anemia. The interaction of these multiple coexisting disease processes is not previously described in the literature. Increased physician awareness of steroid-refractory, Coombs-negative AIHA, and the development of pseudoreticulopenia as a laboratory finding in pernicious anemia may help to improve patient outcomes.

## 1. Introduction

Coombs-negative, steroid-refractory autoimmune hemolytic anemias (AIHAs) are rare, accounting for fewer than 1% of AIHAs. We present a case that meets these criteria and is further complicated by a reduced reticulocyte index despite a functioning bone marrow secondary to newly diagnosed pernicious anemia. A total of 20 cases of simultaneous AIHA and PA are reported in the literature [1]. To our knowledge, this is the first reported case of Coombs-negative, steroid-refractory AIHA with a simultaneous PA.

## 2. Case Presentation

A previously healthy 28-year-old male with the medical history of hypothyroidism presented to an outside hospital with progressive nausea, vomiting, fatigue, exercise intolerance, jaundice, and dark urine for the previous 10 days. Hgb was 3.8 g/dL, and the patient was transferred to an HCA Healthcare Facility in Denver, Colorado, for hematology consultation.

Initial laboratory values were consistent with normocytic hemolytic anemia. Total bilirubin was elevated with a normal direct bilirubin. Haptoglobin was less than 1.0 mg/dL. Lactate dehydrogenase was greater than 600 U/L. MCV was 94.6 fL. Reticulocyte was 3.5% with a reticulocyte index equal to 0.35. Multiple direct and indirect Coombs tests were negative. Peripheral blood smear was reviewed by pathology without pertinent abnormalities. Vitamin B12 was 301 pg/mL. Methylmalonic acid was 187 nmol/L. Homocysteine was 11.3 *μ*mol/L. Three fecal occult blood tests returned negative, and computed tomography imaging was negative for any signs of bleeding. Viral serologies including hepatitis A, B, and C, CMV, EBV, HSV, HIV, and parvovirus were negative. Immunologic work-up including IgG subtype levels, cryoglobulins, rheumatoid factor, cyclic citrullinated peptide, CD55 and CD59, double-stranded DNA, and complement C3 and C4 were all within normal limits. The patient's antiintrinsic factor antibody returned positive. The remainder of the laboratory values was unremarkable. Abdominal ultrasound revealed splenomegaly to 17.6 cm.

Concurrently, the patient was hemolyzing rapidly requiring 1-2 units of packed red blood cells per day. The patient's reduced reticulocyte index prompted investigation for a marrow-mediated process. Abdominal positron emission tomography/computed tomography was negative for focal areas of tracer uptake, and bone marrow biopsy was mildly hypercellular with erythroid hyperplasia and <1% blasts and no overt dysplasia. On hospital day 13, the patient's super-Coombs test returned positive and he was diagnosed with AIHA.

Whereas, the patient was initially treated with high-dose corticosteroids with no improvement. Upon diagnosis of AIHA via the super-Coombs test, the patient was started on 5 consecutive days of intravenous immunoglobulin and weekly infusions of rituximab for 4 weeks. The patient became transfusion-independent following his second dose of rituximab, and he completed his remaining infusions as an outpatient.

## 3. Discussion

AIHA is diagnosed traditionally with a direct antiglobulin Coombs test. At least 90% of AIHAs are Coombs-positive and another 90% will respond to steroids as the first-line treatment [2–5]. The patient of interest demonstrated all of the classic clinical characteristics of AIHA, but his condition was Coombs-negative, steroid-refractory, and had an unexplained reduced reticulocyte index.

The direct antiglobulin test (DAT) “Coombs test” used to confirm AIHA uses a commercial reagent to detect IgG and C3d on the surface of red blood cells [3, 6–9]. Negative tests may be triggered for several reasons, including IgG levels below threshold, an IgA mediated process, or a lack of complement fixation [3–5]. Coombs-negative AIHAs may be evaluated by further serologic testing or treated empirically if suspicion is high enough [5, 7].

Several studies have evaluated the use of flow cytometry (colloquially known as the super-Coombs test) in evaluating DAT-negative cases [6, 7, 10–12]. Flow cytometry-direct antiglobulin testing has a higher sensitivity than the column agglutination technique, the traditional method of conducting a Coombs test [4, 9–11]. A 2012 study found that flow cytometry could detect as few as 30–40 antibody molecules per red blood cell, which is significantly more sensitive than traditional column agglutination DAT that cannot detect values below 500 molecules [3, 10–12].

Despite an eventual diagnosis of AIHA using the super-Coombs test, this case remained a diagnostic conundrum due to an unexplained reduced reticulocyte index. Reduced reticulocyte indexes are associated with AIHAs due to marrow-mediated processes such as leukemias, lymphomas, and aplastic crisis [2, 6]. However, the patient's bone marrow biopsy confirmed appropriate erythroplasia and <1% blasts, consistent with a properly functioning marrow. The disconnection between the central and peripheral findings in the erythroid lineage could not be reconciled with a simple diagnosis of AIHA. Near discharge, an antiintrinsic factor antibody test returned positive. The presence of a simultaneous, newly diagnosed, pernicious anemia (PA) offers as a possible reconciliation for the patient's reduced reticulocyte index in setting of a functioning bone marrow [13, 14]. Of note, the patient's vitamin B12 levels were repleted empirically with no clinical improvement. He also had no history of anemia and did not present with a macrocytosis.

In a state of active hemolysis, the bone marrow releases reticulum-laden reticulocytes to replace lost RBCs. PA marks an exception to this rule; despite an appropriate erythroblastic response of the marrow, the reticulocyte index may remain low [13]. This is due to an alteration in the maturation process of young erythroid cells, such that the ample reticulum is lost prior to exiting the marrow [13]. Consequently, the erythroid cells released by the marrow are not reticulocytes and thus cannot be detected by standard laboratory reticulocyte assays. This altered maturation of red blood cells provides an explanation why, despite his properly functioning bone marrow, this patient had a reduced reticulocyte index [13].

Coexisting PA and AIHA may be stratified into two distinct categories: PA with a transiently positive AIHA and simultaneous primary AIHA and PA. PA with transiently positive AIHA is relatively common and marks low levels hemolysis due to ineffective hematopoiesis in patients with PA [1, 14, 15]. While a Coombs test is often negative, the AIHA will resolve with repletion of vitamin B12 deficiency, and the Coombs test, if initially positive, will turn negative with treatment [13, 14]. Our patient's hemolysis was persistent despite empiric repletion of vitamin B12 on presentation. In AIHA with simultaneous PA, the two processes are independent and the hemoglobin will not recover with vitamin B12 repletion, as it does not address the separate AIHA [14, 15]. Instead, immunosuppression is required for treatment [14]. The autoimmune connection between AIHA and PA is poorly understood. As is typical with other autoimmune disorders, individuals with one autoimmune disorder have a greater susceptibility to develop other autoimmune disorders [1].

Coombs-negative, steroid-refractory AIHAs represent <1% of AIHAs; this finding coupled with a simultaneous, newly diagnosed PA is not previously described in the literature. Key takeaways are that the super-Coombs test should be ordered when suspicion for AIHA remains high despite a negative Coombs test, and a reduced reticulocyte index in the setting of AIHA may not represent inadequacy of erythroid production if there is concomitant PA.

## Data Availability

No data were used to support the study other than reported.
